# Capturing Mechanism and Sustainable Control of Plateau Pika (*Ochotona curzoniae*) Using a Grassland Guidance Trap System on the Qinghai–Tibet Plateau

**DOI:** 10.3390/ani16030491

**Published:** 2026-02-04

**Authors:** Jun Wan, Hong Jin, Jian Yang, Yiming Deng, Xuheng Gao, Yuting Zhou, Weijie Qiao, Wenyong Cai, Haodong Li, Cong Guo, Kun Liu, Xiaodan Wang, Taiping Hou

**Affiliations:** 1 Key Laboratory of Bio–Resource and Eco-Environment of Ministry of Education, College of Life Sciences, Sichuan University, Chengdu 610065, China; junwan88888888@163.com (J.W.); jinhong@scu.edu.cn (H.J.); yjian@swun.edu.cn (J.Y.); ymdeng2716@163.com (Y.D.); xuhenggao@163.com (X.G.); ytzhou0212@163.com (Y.Z.); qiaoweijieyili@163.com (W.Q.); caiwenyong@stu.scu.edu.cn (W.C.); 15319277187@163.com (H.L.); gc6252@sina.com (C.G.); 18980861214@163.com (K.L.); 2 Shiqu Research Station, Sichuan University, Shiqu County 27350, China; 3 Institute of Mountain Hazards and Environment, Chinese Academy of Sciences, Chengdu 610299, China; wxd@imde.ac.cn

**Keywords:** grassland guidance trap system, sustainable control, plateau pika, Qinghai–Tibet Plateau

## Abstract

Infestations of small terrestrial mammals (e.g., rodents and lagomorphs) on the Qinghai–Tibet Plateau pose formidable challenges, which are exacerbated by the region’s vast expanse and extreme environmental conditions. Conventional chemical treatments have proven unsustainable and environmentally detrimental, highlighting the urgent need for innovative solutions. We introduce the Grassland Guidance Trap System (GGTS), an innovative control method specifically designed for ecologically sensitive plateau regions experiencing severe small terrestrial mammal infestations. In this study, we systematically investigated the capturing mechanisms of the GGTS and its sustained control effect during large–scale enclosed field deployment. The results demonstrate that the guide net achieves a 2.62–fold increase in the number of plateau pikas (*Ochotona curzoniae*) captured through targeted behavioral guidance; field–based behavioral trials confirm that the guide net significantly elevates plateau pikas’ observation area entries and observation area duration in adjacent areas. The open–field test demonstrated that the movement distance and activity time of plateau pikas in the corner area of the device were both significantly higher than those in the central area. In addition, rigorous 600–day field tests across 400 hm^2^ confirmed the system’s exceptional efficacy, reducing plateau pika density by 46.38% within 100 m and 20.45% at 500 m. Notably, its effectiveness improved over time, highlighting its potential as a scalable, sustainable management solution. This research not only establishes the GGTS as a viable solution for plateau pika control but also represents a significant advancement in ecological plateau pika management, fostering long–term ecological balance on the Qinghai–Tibet Plateau.

## Simple Summary

On the Qinghai–Tibet Plateau, overpopulated small terrestrial mammals (e.g., rodents and lagomorphs) accelerate grassland degeneration, harming both the environment and people’s livelihoods. Commonly used chemical controls are also damaging and do not offer long–term solutions. To address this, we developed and tested a new, environmentally friendly method—a Grassland Guidance Trap System that uses guide nets to direct small terrestrial mammals toward capture devices. Our results show that these nets effectively attract and channel plateau pika (*Ochotona curzoniae*), making the traps 2.62 times more effective. In a large 600–day field trial, the system worked better the longer it was deployed, reducing plateau pika activity by 46.38% within 100 m and sustaining a 20.45% control rate out to 500 m. This demonstrates that the system is a practical, long–lasting way to manage plateau pika populations. Our work provides a sustainable approach for restoring and protecting the grassland ecosystems of the Qinghai–Tibet Plateau, contributing to the maintenance of ecological balance and health.

## 1. Introduction

The Qinghai–Tibet Plateau, known as the “water tower of Asia” possesses vital natural resources [1]. It plays essential roles in water conservation [2,3], soil preservation [4], climate regulation [5], carbon sequestration [6,7], and biodiversity conservation [8], serving as a crucial ecological security barrier. Nevertheless, alpine grasslands have been experiencing severe degradation in recent years due to escalating overgrazing and climate change impacts [9,10,11]. Combined with limited self–restoration capacity, this degradation creates favorable conditions for infestations of small terrestrial mammals on the Plateau [12,13]. Dominant species include the plateau pika (*Ochotona curzoniae*), plateau zokor (*Eospalax baileyi*), and Himalayan marmot (*Marmota himalayana*). Once their populations grow beyond control, they may exacerbate existing degradation processes [14,15]. In 2017, the total area affected in the Qinghai–Tibet Plateau region (encompassing Qinghai, Xinjiang, Gansu, Tibet, and Sichuan provinces/autonomous regions) reached 22.436 million hectares. Within this, damage related to plateau pikas covered the largest area, reaching 11.52 million hectares. Currently, chemical treatments are the primary management method employed when the density of plateau pikas becomes severely excessive, but they have significant drawbacks: they are difficult to apply on a large scale in alpine regions, have limited effectiveness, and lack long–term sustainability [16].

**Figure 1:**
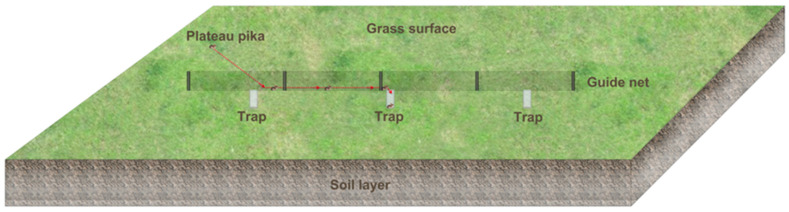
Schematic diagram of the Grassland Guidance Trap System (GGTS).

Achieving sustainable control requires consideration of both the plateau’s unique ecological characteristics and the specific behaviors of the plateau pika species. Physical control methods, particularly trap–fence systems, have garnered attention as promising non-chemical alternatives. The concept has a long history, with early work exploring the use of fences to restrict or guide rodent movements [17]. Subsequent research has further integrated physical barriers, trapping devices, and predator utilization to develop integrated management strategies [18,19,20,21]. However, directly applying these methods to the topographically complex and ecologically fragile alpine grasslands of the Qinghai–Tibet Plateau, which host unique small terrestrial mammals species, still faces significant challenges regarding adaptability, operability, and cost-effectiveness. To address these challenges, our research group developed the Grassland Guidance Trap System (GGTS, Figure 1). The GGTS was specifically optimized for the plateau environment and the behavior of major small terrestrial mammals like the plateau pika. In the 2017 initial trial on artificial grassland, the GGTS efficiently captured multiple small terrestrial mammals species including Blyth’s mountain vole (*Neodon leucurus*), plateau pika, long–tailed dwarf hamster (*Cricetulus longicaudatus*), and Gairdner’s shrewmouse (*Mus pahari*). In the 2019 enclosed plot trial on natural grassland, two and five GGTS units achieved a 42.54% and 52.24% control effect, respectively, over 2 hm^2^ enclosed areas after 30 days of continuous operation. Efficacy progressively increased with extended duration, stabilizing at >60% after 90 days with five units [22]. These results demonstrate the GGTS’s broad applicability and high control efficiency for major small terrestrial mammals across diverse Qinghai–Tibet Plateau grassland ecosystems.

To investigate the mechanisms enabling the GGTS’s efficient capture of plateau pika on Qinghai–Tibet Plateau grasslands and clarify how it exploits behavioral vulnerabilities of this small mammals, while evaluating the GGTS’s efficacy and feasibility at larger implementation scales and over extended durations, this study systematically examined the region’s dominant small terrestrial mammals species, the plateau pika. We first employed high–resolution cameras to document GGTS capture processes and combined this with open–field tests observing the pikas’ activity preference between corner and central areas. Then, we conducted a 600–day continuous monitoring trial within a 400 hm^2^ enclosed test area. This research aims to establish theoretical and methodological foundations for efficient, sustainable, and ecologically sound control targeting excessive densities of plateau pikas on the Qinghai–Tibet Plateau.

## 2. Materials and Methods

### 2.1. GGTS Approach Test for Capturing Plateau Pikas

#### 2.1.1. Test Site

**Figure 2:**
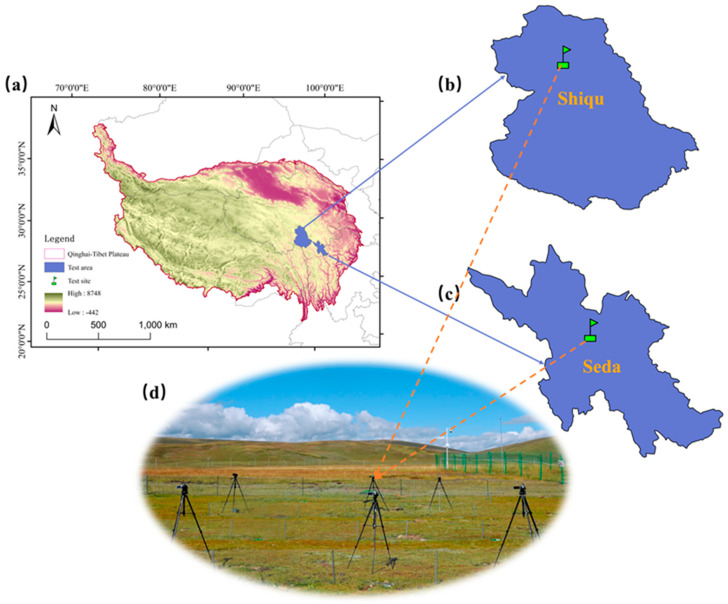
Field Test of the GGTS capture approach for plateau pikas. (**a**–**c**) Location of the test areas and test sites. (**d**) Cameras recording the GGTS process of capturing plateau pikas.

The test sites are situated in natural grassland areas of Xingfu 2nd Village, Seke Town, Seda County, and Guen Village, Derongma Township, Shiqu County, Ganzi Prefecture, China (Figure 2a–c). The coordinates for Xingfu 2nd Village are 32°17′24″ N latitude, 100°18′8″ E longitude, with an altitude of 3923 m. For Guen Village, the coordinates are 33°3′57″ N latitude, 97°57′57″ E longitude, and an altitude of 4156 m (this site was also used for the functional study of the GGTS guide net described in Section 2.2). Vegetation at the sites is dominated by species such as *Kobresia setchwanensis*, *Stipa purpurea*, *Elymus nutans*, *Kobresia humilis*, and *Polygonum viviparum*. The tested animals were the plateau pikas.

#### 2.1.2. Test Design

This test uses high–definition cameras to record the entire process of plateau pikas entering the GGTS traps and analyzes the videos to determine how the GGTS captures the plateau pikas. At the Seda County test site, each GGTS consists of a guide net (20 m in length and 0.50 m in height) and three traps (each trap was constructed from galvanized sheet metal, formed into a cylindrical shape with a diameter of 0.25 m and a depth of 0.50 m). At the Shiqu County test site, each GGTS is composed of a shorter guide net (10 m in length and 0.50 m in height) and three traps (0.25 m in diameter and 0.50 m in depth). After the GGTS installation, we positioned one camera 2 m in front of each trap, aligned vertically with the GGTS guide net. Three cameras were used for simultaneous observation beside each GGTS, and the model of each camera is the SONY-HDRCX900E (Figure 2d). The cameras recorded from 08:00 to 18:00 daily. The Seda test began in July 2018 and lasted for 30 days. The Shiqu test was conducted from June to September 2021, May to November 2022, and June to August 2023. The recorded videos were stored on a hard disk for observation and analysis.

### 2.2. Functional Study of the GGTS Guide Net Under Natural Conditions

#### 2.2.1. Test Design

**Figure 3:**
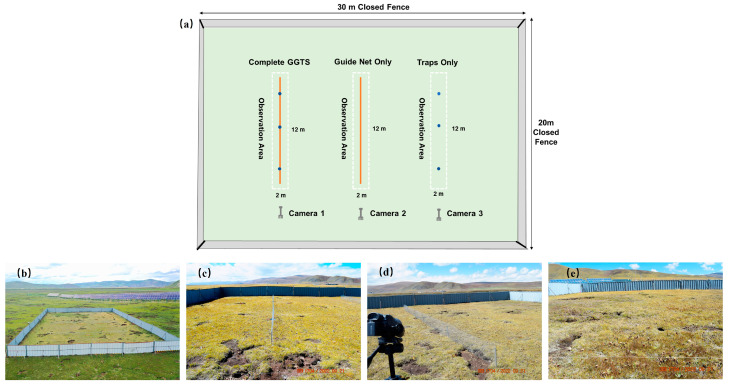
Layout of the test site for the functional trial of the guide net. (**a**) Layout of the device installations in the enclosed test site. (**b**) The enclosed test site. (**c**) The Complete GGTS device. (**d**) The Guide Net Only device. (**e**) The Traps Only device.

A rectangular enclosure, measuring 30 m in length, 20 m in width, and covering an area of 600 m^2^, was installed in the natural grassland using a closed fence. The fence height above ground is 1.50 m, with an underground depth of 0.40 m. Inside the enclosure, three test setups were installed: the Complete GGTS, the Guide Net Only, and the Traps Only (Figure 3a,b). The Complete GGTS consists of a 10 m–long wire mesh with a height of 0.50 m and three traps, each 0.50 m deep and 0.25 m in diameter (Figure 3c). The Guide Net Only setup includes a 10 m–long wire mesh, 0.50 m high (Figure 3d), while the Traps Only setup consists of three traps, each 0.50 m deep and 0.25 m in diameter (Figure 3e). Additionally, a 24 m^2^ observation area (2 m × 12 m) is established around each device.

#### 2.2.2. Test Animals

Prior to installing the test apparatus, 18 healthy adult plateau pikas were released into the enclosure and allowed to acclimate freely for 7 days [23].

#### 2.2.3. Behavioral Observation and Metric Definitions

To quantify the approach and exploratory behavior of plateau pikas toward different test devices and to evaluate the function of the GGTS guide net, this study employed two behavioral metrics: observation area entries and observation area duration. Observation area entries refer to the total number of times plateau pikas fully entered the rectangular observation area (2 m × 12 m) set around each trial device (Complete GGTS, Guide Net Only, and Traps Only) during the observation period. Observation area duration refers to the cumulative total time that plateau pikas spent in the rectangular observation area from entry to exit during the same observation period. These metrics were used to assess the attractive effect of different device configurations and the subsequent lingering behavior of attracted animals around the devices. Behavioral observations were conducted using the Sony HDR-CX900E video cameras (Shanghai Suoguang Electronics Co., Ltd., Shanghai, China). Three cameras were fixedly positioned 2 m in front of each of the three observation areas and recorded continuously from 08:00 to 18:00 daily.

#### 2.2.4. Testing Protocol and Data Recording

The test was conducted in the following sequential phases: (1) Blank Control Phase—prior to the installation of any test apparatus, the “observation area entries” and “ observation area duration” of plateau pikas within the future observation areas corresponding to the three apparatus locations were recorded for 7 consecutive days. (2) GGTS Testing Phase—upon completion of the blank control observations, the three test configurations were installed in the enclosure. Starting the day after installation, the “observation area entries” and “observation area duration” of plateau pikas within the observation area of each configuration were recorded for 7 consecutive days.

These two phases (totaling 14 days) constituted one complete test group. To enhance the robustness of the results, the study repeated the entire process for a total of three complete test groups. After each test group, the original plateau pikas were removed from the enclosure and replaced with 18 new, healthy adult plateau pikas before repeating the entire protocol. The observation methods, recording periods, and data recording procedures remained consistent with the first test group for all subsequent groups.

### 2.3. Movement Trajectory in an Indoor Open-Field Test

#### 2.3.1. Instruments and Measures

Our group utilizes an animal motion trajectory tracking system (EthoVision XT 17.5 system, Noldus Information Technology B.V., Wageningen, Netherlands [24], Supplementary Figure S1) to record the movement distance, speed, activity frequency, and activity time of plateau pikas in different areas of an indoor open–field device [25,26].

**Figure 4:**
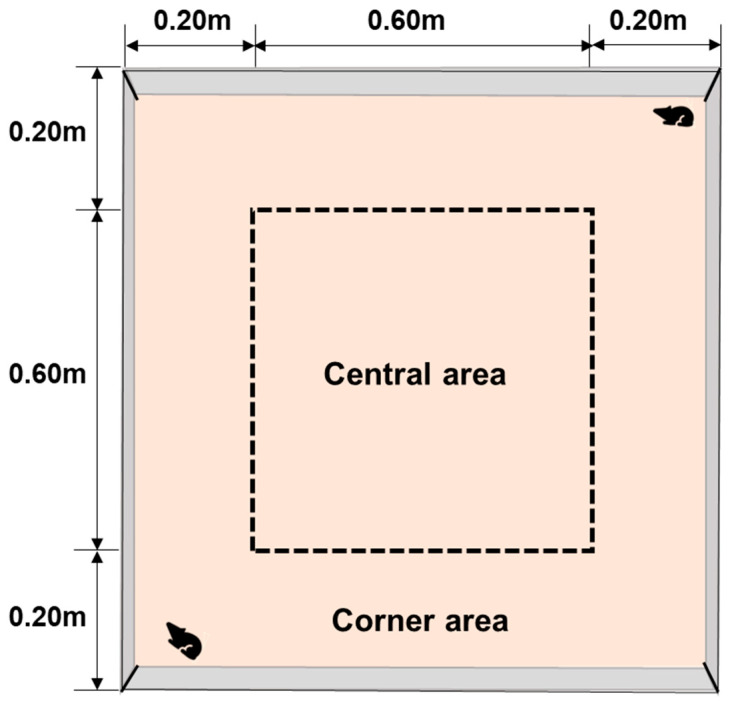
Schematic diagram of the central area and corner area of the indoor open–field device.

The indoor open–field device consists of a closed box with dimensions of 1 m × 1 m × 0.40 m (Supplementary Figure S2). A camera is positioned at the center of the top of the box, and this camera is connected to the animal motion trajectory tracking system to record the plateau pikas’ behavior and movement trajectory in various areas of the open field. The bottom of the box is divided into two main areas: the central area and the corner area. The central area is a square section measuring 0.60 m × 0.60 m, while the remaining space is designated as the corner area. These two areas can be visually distinguished in the tracking system’s recorded images (Figure 4).

#### 2.3.2. Test Procedures

This test employed a standardized open–field paradigm to quantitatively verify whether plateau pikas exhibit a preference for edge areas (wall–following behavior), thereby providing an ethological basis for the guiding function of the field guide net. The plateau pikas used in this experiment were captured using mouse cages in natural grasslands away from the trial area of Derongma Township. Before each trial, disinfectant alcohol was used to clean the feces, urine, and odors from the previous test. The plateau pikas were then placed in the center of the open–field device, and the animal movement trajectory tracking system was activated to record their activity for 10 min (Supplementary Figure S3). After the test, the plateau pikas were returned to the mouse cages and released back into the wild.

### 2.4. Sustained Control in Closed Test Areas

#### 2.4.1. Test Area and Site Description

**Figure 5:**
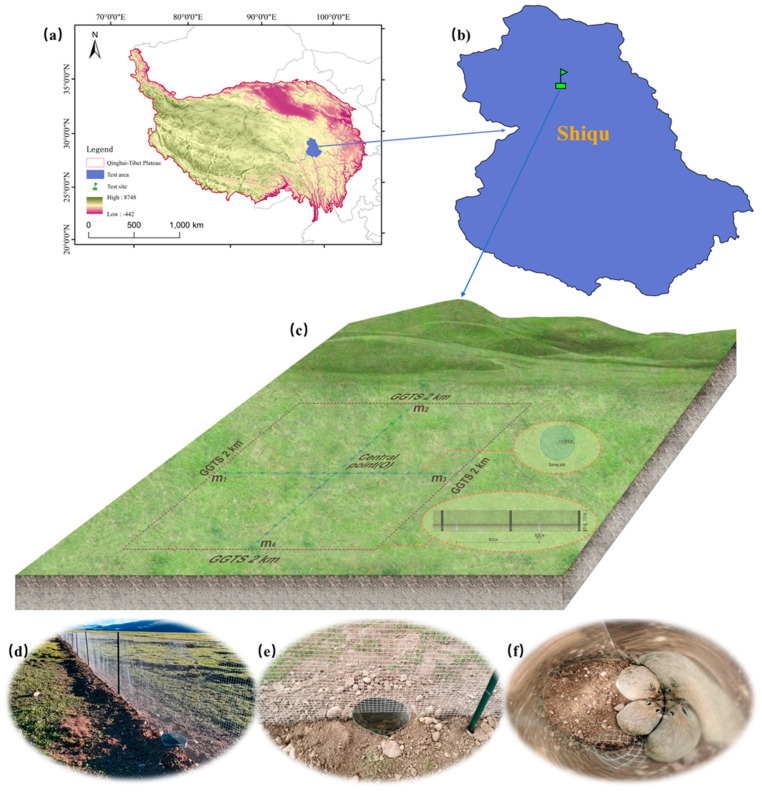
Sustained control trial of the GGTS in an enclosed trial area. (**a**,**b**) Geographical location of the test area and test site. (**c**) Schematic layout of the GGTS installation. (**d**) The GGTS device. (**e**) The GGTS trap. (**f**) The plateau pikas captured in GGTS traps.

This trial was conducted in the natural grassland of Guen Village, Derongma Township, Shiqu County (Figure 5a,b), Sichuan Province (for geographical location and habitat characteristics, see Section 2.1.1). The area had an average active burrow density of 1965/hm^2^, and the plateau pika was the dominant species.

#### 2.4.2. GGTS Design and Installation

An experimental area of 400 hm^2^ was enclosed using the GGTS to prevent the movement and exchange of plateau pikas between the inside and outside of the plot. The GGTS enclosure was square, with a side length of 2 km and a perimeter of 8 km. The fence had an above–ground height of 1.30 m and was buried 0.50 m underground (Figure 5c,d). A total of 267 trap buckets were installed along the fence at 30 m intervals. Each was constructed from galvanized sheet metal and shaped into a cylinder with a diameter of 0.25 m and a depth of 0.50 m (Figure 5e,f). 

#### 2.4.3. Plot Establishment and Population Monitoring

To assess the spatial and temporal efficacy of the GGTS, a total of 37 circular survey plots (radius: 14.6 m; area: 667 m^2^ each) were systematically established within the enclosed area. Plot locations were precisely georeferenced using GPS. They were arranged along transects running perpendicular to the enclosure sides. Starting from the midpoint of each side to center point (O), plots were positioned at 100 m intervals inward, covering distance gradients from 100 m to 1000 m from the fence. Seven additional control plots (BK) were established outside the GGTS enclosure at a minimum distance of 1.5 km, separated by a natural hillside to ensure independence, with 100 m spacing between plots.

The relative population density (PD) of plateau pikas was monitored using the standard active burrow density method. In each plot, all visible burrow entrances were plugged with soil at the beginning of a survey. The number of entrances reopened (i.e., active) after 24 h was counted and served as a proxy for PD.

#### 2.4.4. Survey Schedule

Surveys were conducted over multiple time points to evaluate the sustained control effect: August 2022 (baseline, pre–GGTS installation), followed by post–installation monitoring in September 2022 (30th day), October 2022 (60th day), November 2022 (90th day), March 2023 (180th day), August 2023 (360th day), and May 2024 (600th day).

#### 2.4.5. Calculation of Corrected Control Effect

The sustained control effect of the GGTS was quantified by the corrected active burrow reduction rate (%). This metric is designed to account for background population fluctuations observed in the blank control plots, thereby isolating the population change directly attributable to the GGTS intervention. The calculation was a two–step process: First, the raw control effect for each individual survey plot at a given post–installation time point was calculated as follows:

Control effect (%) = (number of active burrows before GGTS − number of active burrows after GGTS)/(number of active burrows before GGTS) × 100(1)

Subsequently, this raw control effect was corrected using the mean concurrent population change rate from the seven blank control plots to obtain the final efficacy metric.

Corrected control effect (%) = (control effect of a plot − mean control effect of the blank control plots)/(1 − mean control effect of the blank control plots) × 100(2)

The corrected control effect results for plots m_1_–m_4_ are presented in Supplementary Tables S1–S4, separately. The mean corrected control effect refers to the average of four corrected control effects calculated within the same survey period and at the same distance gradient from the m_1_–m_4_ plots.

### 2.5. Tibetan Foxes for Ecological Treatment

#### 2.5.1. Test Site

The test site was located in Xingfu 2 Village, Seke Town, Seda County, Ganzi Prefecture, China, at a longitude of 32°17′24″ N, latitude of 100°18′8″ E, and an altitude of 3923 m. The test duration was from September to November 2018. The site consisted mainly of natural grassland, with species such as *Poa annua*, *Kobresia setchwanensis*, *Stipa purpurea*, *Elymus nutans*, *Kobresia humilis*, and *Polygonum viviparum*. The average number of active burrow in the plot was 2775/hm^2^, and the plateau pika was the dominant small terrestrial mammal species at the site.

#### 2.5.2. Test Design and Observation Methods

**Figure 6:**
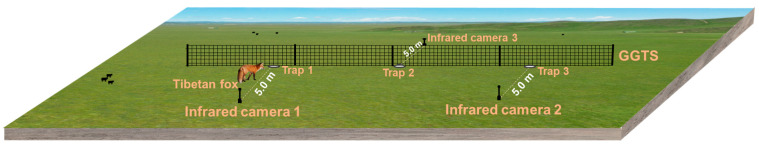
Layout of the monitoring of the Tibetan fox activity around the GGTS.

An infrared camera (H801, Shenzhen Forsafe System Technology Co., Ltd. Shenzhen, China) was positioned near the GGTS for continuous monitoring. The camera recorded the various behaviors of wild Tibetan foxes around the GGTS, enabling an analysis of the ecological significance of the Tibetan foxes for the GGTS (Figure 6 and Supplementary Figure S4).

### 2.6. Data Statistics and Analysis

The data analysis in this experiment was performed using IBM SPSS Statistics 29.0 software. Independent *t*–tests were employed for comparisons between two groups, while one–way ANOVA was utilized for comparisons involving more than two groups (with pairwise comparisons between experimental groups conducted using the LSD method). Graphical representations were created using OriginPro 2025 software.

## 3. Results

### 3.1. GGTS’s Plateau Pikas Capture Mechanism

#### 3.1.1. Capture Approach of the GGTS for Plateau Pikas

**Figure 7:**
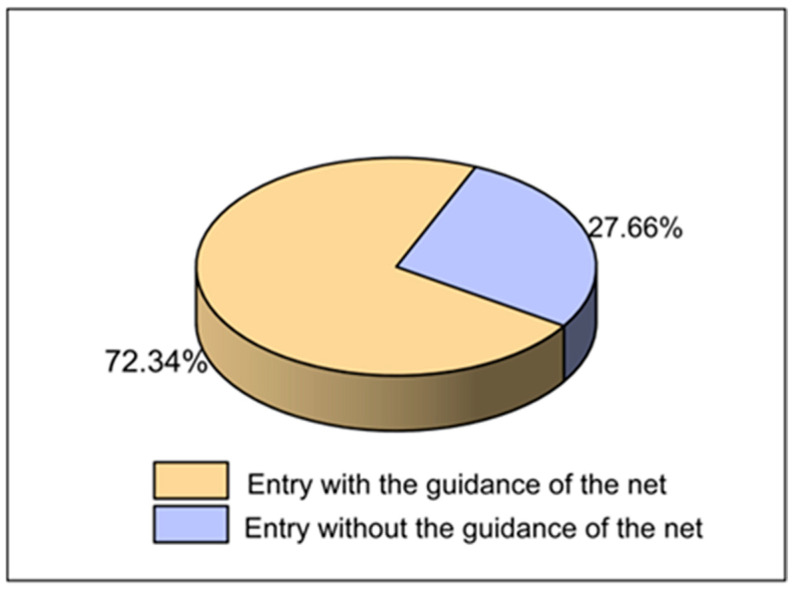
Video analysis results of plateau pikas captured by the GGTS. “Entry with the guidance of the net” refers to the plateau pika falling into the trap with the guidance of the guide net, while “entry without the guidance of the net” refers to the plateau pika falling into the trap without the guidance of the guide net.

**Figure 8:**
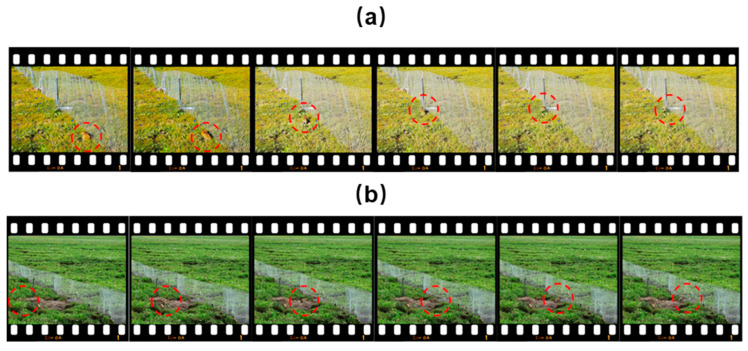
The process of a plateau pika being captured. (**a**) A typical video showing the entire process of a plateau pika being captured under the guidance of the guide net. (**b**) A typical video showing the entire process of a plateau pika being captured without the guidance of a guide net.

To investigate the approach underlying the efficient capture of plateau pikas by the GGTS, we obtained 141 high–resolution videos (4 from Seda County and 137 from Shiqu County) documenting the entire process of plateau pika capture. The results showed that 102 video recordings captured plateau pikas entering traps with the guidance of the guide net, accounting for 72.34% of total capture events (Figure 7). Further analysis identified three main behaviors leading to capture: falling into the trap while running along the net, actively entering the trap while running along the net (Figure 8a, Supplementary Video S1), and falling into the trap during chasing interactions along the net. Additionally, 39 videos showed plateau pikas falling into traps without the guidance of the net, accounting for 27.66% of captures (Figure 7 and Figure 8b, Supplementary Video S2). These findings indicate that captures with the guide net (n = 102) were significantly more frequent than those without it (n = 39) (Chi–square test: *χ^2^* = 28.12, *df* = 1, *p* < 0.001), representing a 2.62–fold increase in capture efficiency attributable to the guide net. We conclude that the GGTS guide net effectively directs moving plateau pikas into traps, which is key to the system’s high capture efficiency.

#### 3.1.2. The Function of the GGTS Guide Net

Attractive and convergent effects of the guide net. As shown in Table 1 and Supplementary Table S5, the number of observation area entries of plateau pikas did not differ significantly between the Traps Only devices (1183.67 ± 121.94 times) and the blank control (1291.33 ± 163.84 times, *p* = 0.840), indicating that traps alone did not significantly influence the entries. In contrast, both the Complete GGTS (2269.33 ± 76.03 times) and the Guide Net Only (2075.67 ± 75.03 times) configurations elicited significantly higher numbers of observation area entries compared with the Traps Only and blank control groups (*p* < 0.001). These results demonstrate that the guide net markedly increases the local activity of plateau pikas, reflecting clear “attractive” and “convergent” effects.

A similar pattern was observed for observation area duration (Table 1; Supplementary Table S6). No significant difference was found between the Traps Only group (591.33 ± 70.01 min) and the blank control (643.33 ± 81.24 min; *p* = 0.976). However, both the Complete GGTS (1187.33 ± 130.64 min) and the Guide Net Only (1120.33 ± 170.93 min) configurations showed significantly longer observation area durations compared with the blank control (*p* < 0.05), further supporting the role of the guide net in prolonging pika presence in the vicinity.

It is seen that the guide net is the key component responsible for the significant increases in both observation area entries and observation area duration around the devices. By exerting distinct attractive and convergent influences, the guide net effectively promotes more frequent and prolonged movement of plateau pikas in its immediate surroundings.

Wall–following behavior and guiding principle. As shown in Table 2, the plateau pikas’ movement distance (5018.57 ± 479.27 cm) and activity time (415.42 ± 29.97 s) in the corner area of the open–field test device were significantly higher than in the central area, where the movement distance was 1077.74 ± 173.59 cm and activity time was 59.20 ± 11.52 s. Plateau pikas moved more frequently in the corner area, likely due to their natural tendency to avoid open spaces. When facing the empty central area, they preferred to move along the “wall–like” features of the corners. Additionally, the plateau pikas’ moving speed in the central area (30.08 ± 5.31 cm/s) was significantly higher than in the corner area (15.52 ± 2.62 cm/s). This suggests that the plateau pika may feel uneasy in the central area and move more quickly to the corner area with its “wall–like features.” These findings further support the idea that plateau pikas prefer to move along the guide net or any “wall–like” structure in the environment, as observed with the Complete GGTS device or the Guide Net Only device.

### 3.2. Sustained Control Effects of GGTS in Closed Test Areas

#### 3.2.1. Progressive Expansion of GGTS Effective Range

Through systematic investigation of changes in active burrows of plateau pikas across all monitoring plots (groups m_1_–m_4_) within an enclosed trial area, this study evaluated the sustained control effect of the GGTS. Long–term observations indicate that as the operating time of the GGTS increases, its effective area of influence progressively expands outward from the vicinity of the installation. As shown in Table 3, during the 30-day intervention phase, the system achieved 18.31% efficacy at 100 m along vertical transects, significantly surpassing distal performance (200–1000 m range). By day 60, efficacy dominance shifted radially outward to 200 m (16.06%), exceeding values at 300–1000 m. Sustained operation until day 180 enhanced 200 m efficacy to 19.74%, contrasting sharply with diminished effects at 300–1000 m. After 600 days of continuous deployment, maximal effective distance extended to 500 m (20.45%), while distal zones (700–1000 m) exhibited negative efficacy. The temporal trajectory demonstrated progressive expansion of GGTS’s operational range from 100 m (18.31% efficacy at day 30) to 500 m (20.45% at day 600), confirming enhanced spatial penetration with prolonged intervention.

#### 3.2.2. Progressive Improvement in GGTS Control Effect at Same Distances

**Figure 9:**
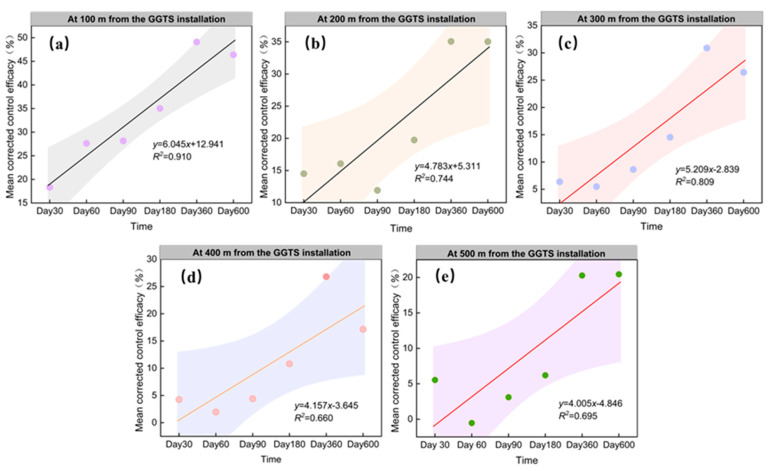
Relationship between the mean corrected control effect of survey plots and the duration of GGTS—based control: (**a**) 100 m from the GGTS; (**b**) 200 m from the GGTS; (**c**) 300 from the GGTS; (**d**) 400 from the GGTS; and (**e**) 500 m from the GGTS.

**Figure 10:**
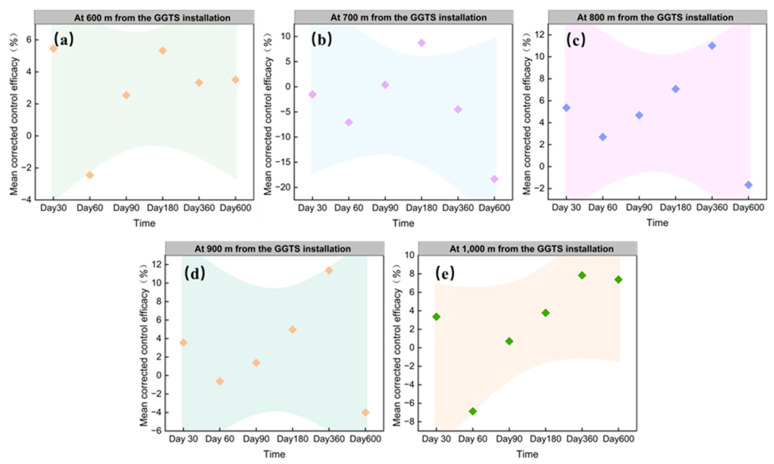
Relationship between the mean corrected control effect of survey plots and the duration of GGTS—based control: (**a**) 600 m from the GGTS; (**b**) 700 m from the GGTS; (**c**) 800 m from the GGTS; (**d**) 900 m from the GGTS; and (**e**) 1000 m from the GGTS.

This experiment systematically monitored the control effect across survey plots (m_1_–m_4_ group) over sustained intervention periods and statistically analyzed the mean corrected efficacy at equivalent distances, thereby elucidating the dynamic relationship between GGTS–mediated control effects and prolonged implementation duration under identical distance conditions. The results demonstrated that during the 600–day GGTS—mediated plateau pika control period, the system exhibited progressive efficacy enhancement, corresponding to prolonged intervention duration at the same distances. Specifically, at 100 m from the installation, efficacy increased significantly over time (*y* = 6.045*x* + 12.941; *R^2^* = 0.910, Figure 9a), with measured values of 18.31%, 27.63%, 28.16%, 35.02%, 49.08%, and 46.38% on days 30, 60, 90, 180, 360, and 600, respectively. Progressing outward, at 200 m (*y* = 4.783*x* + 5.311; *R*^2^ = 0.744, Figure 9b), 300 m (*y* = 5.209*x* − 2.839; *R*^2^ = 0.809, Figure 9c), 400 m (*y* = 4.157*x* − 3.645; *R*^2^ = 0.660, Figure 9d), and 500 m (*y* = 4.005*x* − 4.846; *R*^2^ = 0.695, Figure 9e), consistent upward trends were observed with differential regression slopes. Beyond 500 m, a threshold effect emerged: no significant linear relationships were detected between efficacy and control duration at 600–1000 m distances (Figure 10a–e).

Consequently, as the duration of GGTS implementation in grasslands increases, the effective control distance (coverage radius) for plateau pika progressively expands. Concurrently, at fixed distances from the installation, the control effect demonstrates progressive enhancement corresponding to extended operational duration. This dual mechanism achieves sustained and effective suppression of plateau pika population density in the experimental areas, demonstrating the feasibility of long–term, continuous, and large–scale deployment of GGTSs for grassland plateau pika management.

## 4. Discussion

### 4.1. Prospects for Sustainable Control of Plateau Pikas with the GGTS

Plateau pikas are vital components of the grassland ecosystem on the Qinghai–Tibet Plateau [27,28], playing a crucial role in maintaining food chain balance. However, when natural or anthropogenic factors lead to their overabundance in localized areas, they can adversely affect grassland ecological health, necessitating the implementation of scientifically sound and effective management measures. Although conventional chemical control methods remain widely used, they suffer from fundamental limitations and their efficacy typically lasts only 1–2 years [13,29], failing to prevent rapid population recovery, which is often compounded by compensatory population growth or enhanced pesticide resistance. Furthermore, the vast territory, harsh environment, and sparse human population of the Qinghai–Tibet Plateau make repeated large–scale chemical applications neither feasible nor sustainable [30]. Additional ecological risks include environmental contamination and non–target organism mortality [31,32,33]. Consequently, there is an urgent need to develop novel control strategies for plateau landscapes that are ecologically friendly, durable, and scalable.

To address this challenge, our research group developed the GGTS for plateau pika management. This study conducted a 600–day continuous monitoring in a 400 hm^2^ enclosed experimental area located in Shiqu County. We observed progressive enhancement of plateau pika suppression efficiency with prolonged GGTS deployment duration, maintaining relatively stable control effects over a two–year period. Notably, the system exhibited an expanding control effect radius over time, with current data indicating significant suppression within a 500 m unidirectional range from the GGTS units. Importantly, this approach achieves balanced population regulation that prevents both mass outbreaks and ecological risks of population eradication. The system’s persistent functionality (multi–year efficacy post–installation) overcomes the limitations of conventional chemical methods regarding transient effects and scalability constraints, enabling sustainable large-area implementation. Ongoing investigations focus on quantifying maximum effective distances, with preliminary evidence suggesting that natural plateau pika migration patterns may further extend the GGTS’s operational range over time.

This study employed enclosed fencing to prevent plateau pikas from migrating into or out of the experimental area, thereby minimizing the confounding effect of individual movement on population dynamics and allowing for a more precise evaluation of the inherent control effect of the GGTS. To further distinguish the intervention effect of GGTSs from background factors, such as natural fluctuations and seasonal variations [34,35,36], seven blank control plots were established outside the enclosure, and a correction formula was applied to the collected data. All control effect values presented in this study have been adjusted accordingly, effectively removing background population fluctuations driven by climate, resource availability, and seasonal cycles, thus clearly demonstrating the actual control performance of the GGTS on plateau pikas. In addition, the sustainability of GGTS—mediated control remains a key focus. The system showed a high capture rate shortly after installation, which gradually declined over time. This reduction is attributed mainly to two factors: (1) the initially high population density led to greater capture success, but as continued control reduced plateau pika numbers, capture rates decreased accordingly, with control effectiveness peaking and stabilizing after several months; and (2) it is hypothesized that earlier captured individuals may have influenced the behavior of later plateau pikas.

The GGTS is a purely physical control method that causes no pollution to the grassland ecosystem. This technology transforms traditional plateau pika control into sustainable, long-term population management, representing a significant conceptual and strategic breakthrough. The system enables continuous suppression of populations, facilitating the establishment of a continuous and controllable management model at the population level. In practical applications, GGTS parameters can be optimized according to local small terrestrial mammals’ density and geographical conditions. Integrated with grassland restoration techniques such as grazing exclusion, it can form a comprehensive management framework for degraded alpine grasslands. This provides a new paradigm for addressing the challenges of plateau pika overabundance and associated grassland degradation on the Tibetan Plateau, playing a critical role in safeguarding this vital ecological security barrier.

### 4.2. Ecological Synergistic Processing Role of Tibetan Foxes in GGTS—Controlled Plateau Pika

**Figure 11:**
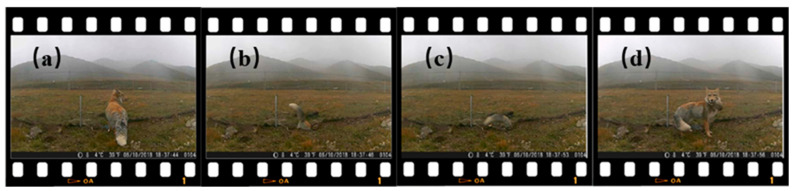
Video photos of the Tibetan fox capturing a plateau pika in the GGTS trap. (**a**) The Tibetan fox observes the GGTS trap. (**b**,**c**) The Tibetan fox attempts to remove the plateau pika from the GGTS trap. (**d**) The Tibetan fox successfully removes the plateau pika from the GGTS trap.

As an apex predator in the food chain of the Qinghai–Tibet Plateau, the Tibetan fox is the most common canid species in the alpine steppe ecosystem. Occupying a top trophic position, it plays a critical role in maintaining biodiversity [37], with the plateau pika serving as its primary prey [38]. In this study, the GGTS effectively captured pikas, and we observed that the trapped plateau pikas became part of the food chain for Tibetan foxes. During the first 30 days after system installation, infrared camera monitoring recorded 21 independent events of Tibetan foxes successfully retrieving pikas from the traps, along with 96 instances of foxes exploring or moving near the traps. Further infrared monitoring showed that when Tibetan foxes detected pikas in the traps, they attempted to enter and capture them (Figure 11, Supplementary Video S3). Notably, not all captured pikas were immediately consumed by Tibetan foxes. The GGTS is a physical barrier device that does not directly kill plateau pikas; its main function is to create predation opportunities for foxes and other predators, while also allowing for other outcomes such as manual removal. These findings indicate that our approach goes beyond the traditional view of simply treating plateau pikas as “pests” to be eliminated, and instead positions the GGTS as an ecological management tool for “high density population regulation—resource redistribution.” By locally reducing excessive pika densities in degraded grasslands and channeling the captured biomass upward through the food chain, the GGTS provides an important supplementary food source for Tibetan foxes. Therefore, the GGTS not only helps support the stability of predator populations but also promotes the restoration of degraded grassland ecosystems, demonstrating significant ecological value.

## 5. Conclusions

This study documented the complete process of plateau pika capture by the GGTS through video recordings and observed behavioral differences among pikas around different devices. Video analysis revealed that the guide net significantly increased observation area entries (2269.33 ± 76.03 vs. 1183.67 ± 121.94 times, *p* < 0.001) and observation area duration (1187.33 ± 130.64 vs. 591.33 ± 70.01 min, *p* < 0.05) around the device, generating “attractive” and “convergent” effects on plateau pikas. Consequently, captures with the guide net were 2.62 times those achieved without its guidance. Additionally, we conducted large–scale field tests and observed that as the duration of GGTS application increased, its control effect on grassland pikas improved. After 600 days, the GGTS maintained a control effect of 20.45% at 500 m, successfully regulating pika population density within the defined area. This study establishes the GGTS as a sustainable and scalable technical approach for controlling plateau pikas in alpine grasslands.

## Figures and Tables

**Table 1:** Observation area entries and observation area duration of plateau pikas across different experimental configurations.

Devices	Observation Area Entries (Times)	Observation Area Duration (min)
Complete GGTS	2269.33 ± 76.03 a	1187.33 ± 130.64 a
Guide Net Only	2075.67 ± 75.03 a	1120.33 ± 170.93 a
Traps Only	1183.67 ± 121.94 b	591.33 ± 70.01 b
Blank Control	1291.33 ± 163.84 b	643.33 ± 81.24 b

Note: blank control corresponds to the observation area prior to device installation; “observation area entries” and “observation area duration” are behavioral indicators used to explain the function of the GGTS guidance net. Different lowercase letters indicate significant differences (*p* < 0.05).

**Table 2:** Activity patterns of plateau pikas in different areas of the open-field device.

Activity Area	Movement Distance (cm)	Movement Speed (cm/s)	Activity Frequency (Times)	Activity Time (s)
Central area	1077.74 ± 173.59 a	30.08 ± 5.31 a	22.65 ± 4.24 a	59.20 ± 11.52 a
Corner area	5018.57 ± 479.27 b	15.52 ± 2.62 b	37.38 ± 6.75 a	415.42 ± 29.97 b

Note: different lowercase letters indicate significant differences (*p* < 0.05).

**Table 3:** Mean corrected control effect of GGTS at different implementation times.

Distance from GGTS (m)	Mean Corrected Control Effect (%)					
	Day 30	Day 60	Day 90	Day 180	Day 360	Day 600
100	18.31	27.63	28.16	35.02	49.08	46.38
200	14.51	16.06	11.92	19.74	35.06	35.03
300	6.38	5.49	8.64	14.54	30.88	26.43
400	4.25	1.97	4.41	10.82	26.81	17.16
500	5.53	−0.54	3.10	6.20	20.28	20.45
600	5.46	−2.45	2.54	5.33	3.33	3.51
700	−1.52	−7.07	0.38	8.74	−4.51	−18.33
800	5.36	2.70	4.68	7.07	11.01	−1.67
900	3.56	−0.63	1.38	4.96	11.36	−3.99
1000	3.36	−6.87	0.71	3.78	7.84	7.38

## Data Availability

The data presented in this study are available on request from the corresponding author.

## Supplementary Materials

The following supporting information can be downloaded at: https://www.mdpi.com/article/10.3390/ani16030491/s1, Figure S1: The Noldus EthoVision XT system used to test the movement trajectory of plateau pikas; Figure S2: Indoor open field test apparatus; Figure S3: Heat map of the activity trajectory of plateau pikas in the open field device; Figure S4: Tibetan fox frequently active around GGTS; Table S1: Control effect of GGTS on rodents at different distances from Group m_1_ at different implementation times; Table S2: Control effect of GGTS on rodents at different distances from Group m_2_ at different implementation times; Table S3: Control effect of GGTS on rodents at different distances from Group m_3_ at different implementation times; Table S4: Control effect of GGTS on rodents at different distances from Group m_4_ at different implementation times; Table S5: Recording the number of plateau pika entries into the observation areas of different devices; Table S6: Recording the time spent of plateau pika entries into the observation areas of different devices; Video S1: Plateau pika actively entered the trap while running along the net; Video S2: Plateau pika entered into the trap without the guidance of the net; Video S3: Tibetan fox captured the plateau pika in the trap.
