# Supplementary material for: Capturing Mechanism and Sustainable Control of Plateau Pika (Ochotona curzoniae) Using a Grassland Guidance Trap System on the Qinghai–Tibet Plateau
Source: Animals (Basel). 2026 Feb 4;16(3):491. doi: 10.3390/ani16030491 (PMC12896900; doi:10.3390/ani16030491)
Supplement: Supplementary file 1 [file animals-16-00491-s001.zip › Supplementary Information.pdf]

Supplementary Information for

Capturing Mechanism and Sustainable Control of Plateau Pika *(Ochotona curzoniae)* using a Grassland Guidance Trap System on the Qinghai–Tibet Plateau

Jun Wan[1,2], Hong Jin[1,2], Jian Yang[1], Yiming Deng [1], Xuheng Gao[1], Yuting Zhou[1,2], Weijie Qiao[1,2], Wenyong Cai[1,2], Haodong Li[1,2], Cong Guo[1], Kun Liu[1,2], Xiaodan Wang[3], Taiping Hou[1,2 *]

[1] Key Laboratory of Bio-Resource and Eco-Environment of Ministry of Education, College of Life Sciences, Sichuan University, Chengdu, 610065, China; junwan88888888@163.com (J.W.); jinhong@scu.edu.cn (H.J.); yjian@swun.edu.cn (J.Y.); ymdeng2716@163.com (Y.D.); xuhenggao@163.com (X.G.); YtZhou0212@163.com (Y.Z.); qiaoweijieyili@163.com (W.Q.); caiwenyong@stu.scu.edu.cn (W.C.); 15319277187@163.com (H.L.); gc6252@sina.com (C.G.); 18980861214@163.com (K.L.)

[2] Shiqu Research Station, Sichuan University, Ganzi Prefecture, 627350, China

[3] Institute of Mountain Hazards and Environment, Chinese Academy of Sciences, Chengdu, 610299, China; wxd@imde.ac.cn (X.W.)

* Corresponding author: Taiping Hou, e-mail: houtp@scu.edu.cn

Figures and Table legends

Figure S1. The Noldus EthoVision XT system used to test the movement trajectory of plateau pikas.

Figure S2. Indoor open field test apparatus.

Figure S3. Heat map of the activity trajectory of plateau pikas in the open field device.

Figure S4. Tibetan fox frequently active around GGTS.

Table S1. Control effect of GGTS on rodents at different distances from Group m_1 at different implementation times.

Table S2. Control effect of GGTS on rodents at different distances from Group m_2 at different implementation times.

Table S3. Control effect of GGTS on rodents at different distances from Group m_3 at different implementation times.

Table S4. Control effect of GGTS on rodents at different distances from Group m_4 at different implementation times.

Table S5. Recording the number of plateau pika entries into the observation areas of different devices.

Table S6. Recording the time spent of plateau pika entries into the observation areas of different devices.

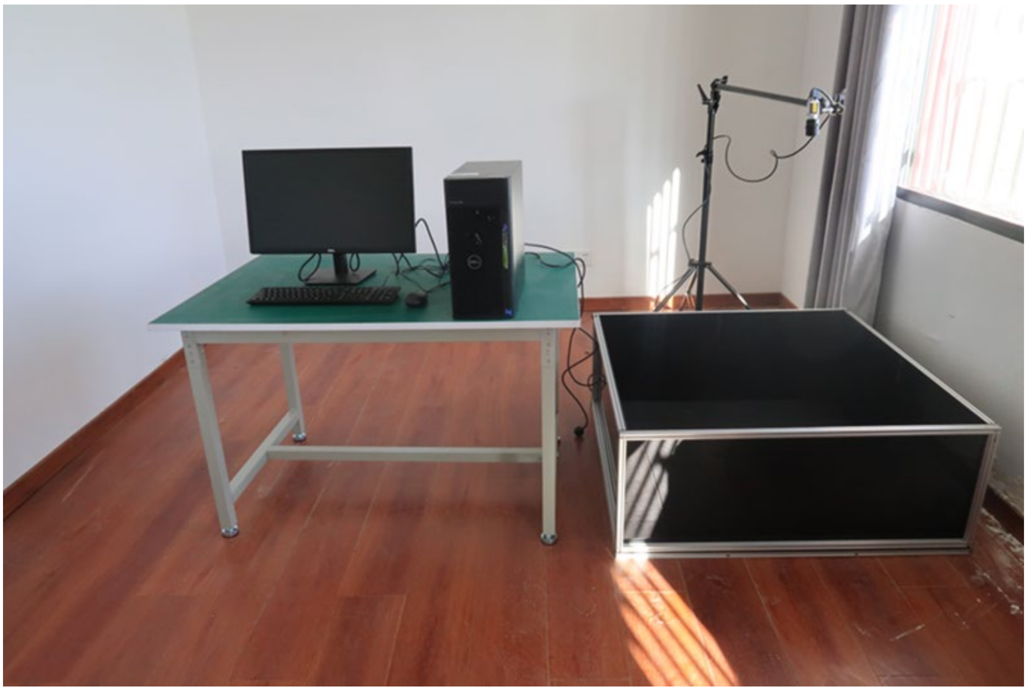

Figure S1. The Noldus EthoVision XT system used to test the movement trajectory of plateau pikas.

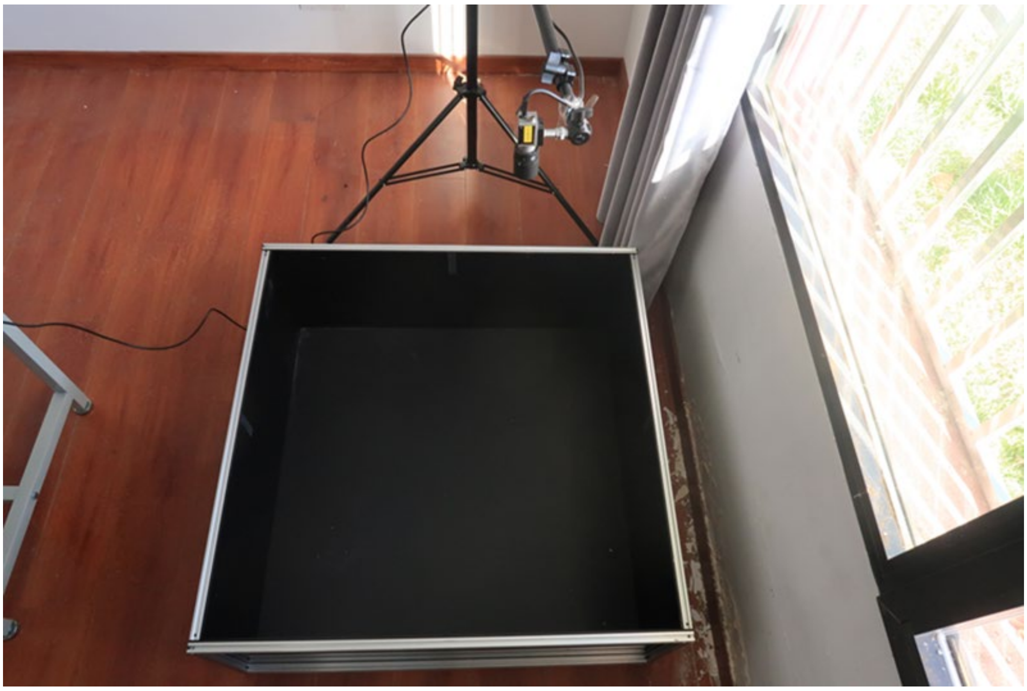

Figure S2. Indoor open field test apparatus.

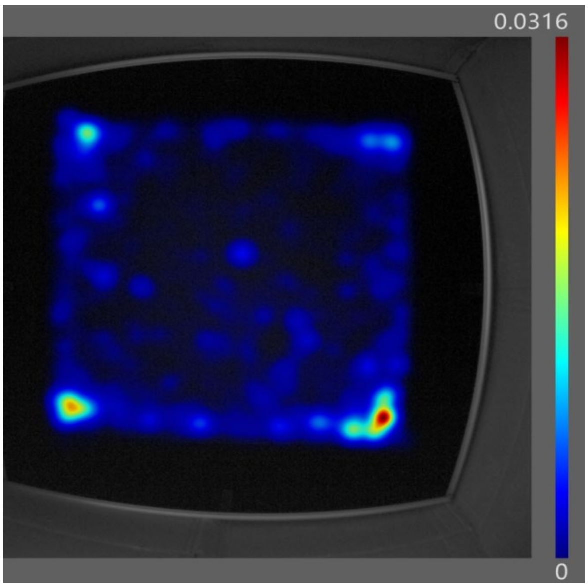

Figure S3. Heat map of the activity trajectory of plateau pikas in the open field device. The redder the color, the longer the plateau pika remains in that area, while the bluer the color, the shorter the stay.

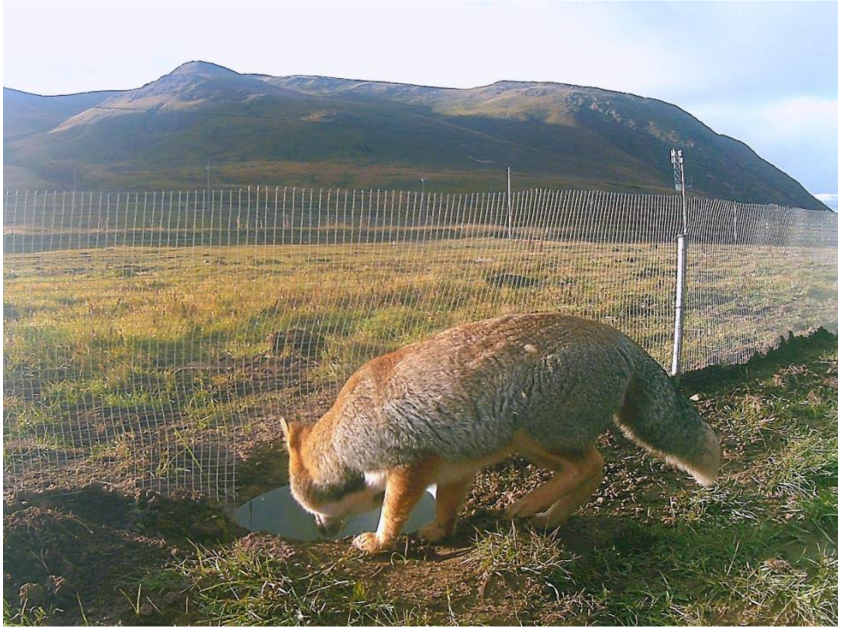

Figure S4. Tibetan fox frequently active around GGTS.

Table S1. Control effect of GGTS on rodents at different distances from Group m_1 at different implementation times.

Plots	Distance (m)	Corrected control effect（%）					
		Day 30	Day 60	Day 90	Day 180	Day 360	Day 600
Point O	1,000	3.36	-6.87	0.71	3.78	7.84	7.38
1	900	2.05	-3.52	1.07	3.32	-1.26	-11.26
2	800	2.31	4.31	6.52	6.09	29.35	5.51
3	700	-21.99	-16.71	11.7	23.18	-23.91	-34.64
4	600	-0.28	0.41	1.56	3.96	-2.10	-3.85
5	500	10.29	-10.20	0.36	10.87	28.71	27.34
6	400	5.72	9.79	7.37	7.82	39.18	20.88
7	300	-2.88	0.50	8.14	15.48	47.35	31.28
8	200	12.34	-2.32	6.16	16.15	24.45	17.33
9	100	11.27	18.65	20.65	32.18	54.54	49.85

Table S2. Control effect of GGTS on rodents at different distances from Group m_2 at different implementation times.

Plots	Distance (m)	Corrected control effect（%）					
		Day 30	Day 60	Day 90	Day 180	Day 360	Day 600
Point O	1,000	3.36	-6.87	0.71	3.78	7.84	7.38
1	900	5.86	-5.21	2.55	6.74	3.76	-24.26
2	800	8.71	7.03	6.43	12.71	13.44	0.70
3	700	6.43	-9.32	-15.32	-3.00	-20.01	-21.54
4	600	9.98	-8.29	3.52	4.57	7.84	12.46
5	500	8.28	-2.51	-1.67	-0.47	27.58	49.80
6	400	12.14	19.44	24.80	26.73	37.12	21.00
7	300	9.40	-4.25	-1.29	3.03	21.16	48.17
8	200	17.12	26.95	-8.44	5.53	40.26	41.23
9	100	23.76	34.74	33.99	38.08	45.04	41.95

Table S3. Control effect of GGTS on rodents at different distances from Group m_3 at different implementation times.

Plots	Distance (m)	Corrected control effect（%）					
		Day 30	Day 60	Day 90	Day 180	Day 360	Day 600
Point O	1,000	3.36	-6.87	0.71	3.78	7.84	7.38
1	900	7.18	3.88	2.18	9.50	15.19	18.57
2	800	2.75	5.19	6.91	8.39	2.52	-16.32
3	700	6.74	-4.21	-0.03	1.50	3.29	-10.55
4	600	5.92	5.15	7.11	10.30	25.13	2.55
5	500	5.29	8.66	11.90	12.03	2.11	-7.35
6	400	-10.78	-9.35	-12.28	8.36	11.50	7.34
7	300	9.01	13.08	14.61	20.04	29.80	19.52
8	200	16.11	21.48	27.44	30.90	37.22	41.74
9	100	20.23	34.67	32.92	40.43	44.67	47.26

Table S4. Control effect of GGTS on rodents at different distances from Group m_4 at different implementation times.

Plots	Distance (m)	Corrected control effect（%）					
		Day 30	Day 60	Day 90	Day 180	Day 360	Day 600
Point O	1,000	3.36	-6.87	0.71	3.78	7.84	7.38
1	900	-0.87	2.33	-0.29	0.29	27.73	1.01
2	800	7.67	-5.75	-1.16	1.09	-1.27	3.42
3	700	2.73	1.95	5.18	13.26	22.61	-6.6
4	600	6.23	-7.08	-2.03	2.48	-17.55	2.86
5	500	-1.75	1.89	1.82	2.37	22.71	12.01
6	400	9.91	-12.01	-2.27	0.38	19.44	19.41
7	300	9.98	12.64	13.10	19.62	25.21	6.74
8	200	12.46	18.13	22.53	26.39	38.30	39.80
9	100	17.99	22.47	25.08	29.39	52.05	46.45

Table S5. Recording the number of plateau pika entries into the observation areas of different devices.

Devices	observation area entries (times)			
	Repetition Group 1	Repetition Group 2	Repetition Group 3	Mean ± SD
Complete GGTS	2,326	2,177	2,305	2,269.33 ± 76.03 a
Guide Net Only	1,984	2,109	2,134	2,075.67 ± 75.03 a
Traps Only	1,301	1,059	1,191	1,183.67 ± 121.94 b
Blank Control	1,104	1,355	1,415	1,291.33 ± 163.84 b

Note: The lowercase letters indicate significant differences ($P < 0.05$)

Table S6. Recording the time spent of plateau pika entries into the observation areas of different devices..

Devices	Observation area duration (min)			
	Repetition Group 1	Repetition Group 2	Repetition Group 3	Mean ± SD
Complete GGTS	1,293	1,044	1,225	1,187.33 ± 130.64 a
Guide Net Only	1,104	1,299	958	1,120.33 ± 170.93 a
Traps Only	517	651	606	591.33 ± 70.01 b
Blank Control	701	682	547	643.33 ± 81.24 b

Note: The lowercase letters indicate significant differences ($P < 0.05$)
